# Proposal for preventing fatal nitrogen gas inhalation while working: an autopsy case and review of the literature

**DOI:** 10.1265/ehpm.26-00143

**Published:** 2026-10-21

**Authors:** Arisa Takeda, Kenshiro Aiko, Marin Takaso, Mami Nakamura, Masahito Hitosugi

**Affiliations:** Department of Legal Medicine, Shiga University of Medical Science, Otsu, Japan

**Keywords:** Fatal nitrogen gas inhalation, Industrial accident, Autopsy case, Double-check system

## Abstract

**Background:**

Nitrogen is a colorless, odorless, and generally inert gas that acts as a simple asphyxiant by displacing oxygen. While accidental nitrogen gas inhalation typically occurs in confined spaces, fatalities resulting from mask misconnections during industrial work are extremely rare. Inhaling excess nitrogen can rapidly cause disorientation, rendering victims unable to escape critical situations. Therefore, developing preventive measures is an urgent priority.

**Case presentation:**

A Japanese factory worker in his 40s was found in cardiopulmonary arrest inside a pot he was cleaning alone and subsequently died. The company’s standard protocol requires a two-person team. In poor ventilation, workers typically use face masks connected to an atmospheric fresh air line or an oxygen source to meet physical demands. The victim was discovered wearing a mask mistakenly connected to a nitrogen gas line located adjacent to the oxygen line. Although both gas sources were color-coded and labeled, this misconnection occurred. After a forensic autopsy, his cause of death was diagnosed as nitrogen poisoning.

**Discussion:**

A literature review spanning PubMed data up to 2024 identified only 17 unintentional nitrogen-related deaths, with only 5 involving mask misconnections, mostly due to poor labeling. Japanese labor statistics from 1993 to 2022 recorded 30 nitrogen-related fatalities, including 4 mask-related cases, although detailed medical data were absent. To our knowledge, this case represents the first detailed forensic autopsy report in the English literature of a worker dying from nitrogen poisoning due to a mask misconnection. Our findings suggest that relying solely on the single person’s visual information is insufficient to prevent fatal human error.

**Conclusion:**

To diminish work-related nitrogen fatalities, we propose two potential preventive measures. First, to actively prevent human errors, we propose several technical approaches for fail-safe designs, such as incompatible or dedicated connectors, separation of supply systems, and equipping masks with oxygen monitoring devices that automatically shut off the gas supply when the oxygen concentration falls below a safe threshold. Secondary, a strict “double-check” system must be implemented. Workers should never connect air lines alone. The other worker must verify the connection and remain available to remove the mask and perform immediate resuscitation if an accident occurs.

## Background

Nitrogen is a colorless, odorless nontoxic, and generally inert gas that comprises 78% of the air. Nitrogen is considered to be a simple asphyxiate gas because the displacement of nitrogen from oxygen causes a life-threatening condition. A reduction in atmospheric oxygen to less than 25% of normal composition can produce unconsciousness in seconds and death within minutes. Therefore, suicide using nitrogen gas has often occurred [1]. Additionally, accidental deaths by inhaling nitrogen gas have been reported [1–6].

Regarding cases of accidental death due to nitrogen, the most common situation is workers who suffered from exposure of nitrogen gas in a closed environment. There have been a few autopsy reports of such deaths [1–8]. In other situations, the victims had inhaled nitrogen gas via a face mask that was connected to gas sources. One of the major situations of nitrogen-related accidents is scuba diving. Nitrogen is an anesthetic gas with euphoric and narcotic effects. While scuba diving, air is supplied at increased pressure at depth, resulting in nitrogen being progressively dissolved in plasma and tissues according to its partial pressure. This situation can result in nitrogen narcosis, in which disorientation and impaired judgment resembling ethanol intoxication occur, and this can lead to death due to drowning [8]. Other accidental inhalations of nitrogen via a face mask have occurred during work [7]. In these cases, the mask was mistakenly connected to nitrogen gas, not air or oxygen [7]. Once nitrogen gas is aspirated via a mask, escaping from such a situation is difficult owing to the narcotic effects, and victims promptly become critical. Therefore, effective measures to prevent accidental nitrogen gas inhalation via a face mask should be promoted.

We report a fatal case of nitrogen gas poisoning in which the victim had mistakenly connected his mask to the line of a nitrogen gas source. To our knowledge, this is the first case report presenting detailed forensic autopsy findings (external examination, internal findings, histopathology, and toxicology) of such a fatal accident during work in the English literature. After reviewing the literature, we propose effective preventive measures that could contribute to a reduction in work-related fatalities.

## Case presentation

A Japanese man in his 40s was found in cardiopulmonary arrest by a pot at a factory. He had engaged in the cleaning of the interior of the pot by himself. According to the local rules, the cleaning of pots needs to be performed by a pair of workers. Sufficient air is not supplied to the interior of the pot. Therefore, workers usually use a face mask, which uses fresh air of the atmosphere. Moreover, workers sometimes connect a line to an oxygen source instead of aspirating atmospheric air because of the elevated demand for oxygen with increased activity. When the victim was found, he wore a face mask with a line connected to a nitrogen gas source. The nitrogen gas source was located next to an oxygen gas source. A label was attached on each source and specified nitrogen in yellow and oxygen in blue (Fig. 1).

**Fig. 1:**
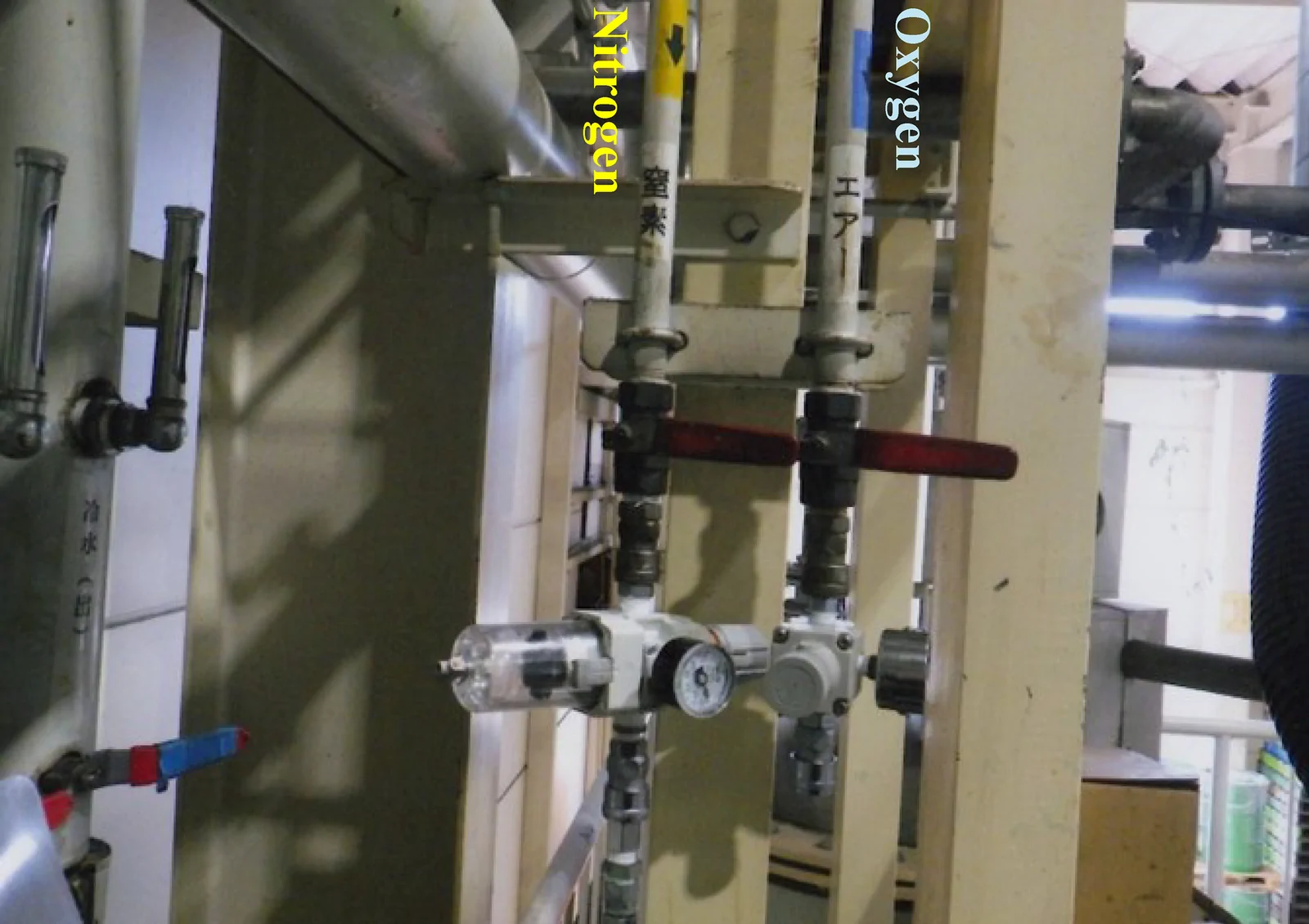
A yellow label marked nitrogen gas, while a blue label marked oxygen (actual accident site).

Immediately after the victim was found, cardiopulmonary resuscitation was performed. Although he was transported to the hospital, he was pronounced dead 30 minutes after being found. According to his kin, he had no previous medical history or medications.

At a forensic autopsy, no external injuries were found. Petechial hemorrhages were found on the palpebral conjunctiva and inner scalp. Dark red fluid blood was found in the heart cavity. No abnormal findings were found in the viscera except for congestion. Histopathologically, fatty liver and acute kidney injury were observed. Toxicologically, alcohol or other drugs were not determined.

Because the measurement of nitrogen gas from the cadaver’s blood was technically difficult, the presence of the gas in his body could not be proved. The victim revealed acute death findings such as petechial hemorrhages, dark red fluid blood in the heart cavity and visceral congestion. Neither internal diseases nor injuries that could contribute to his death, nor findings suggestive of other intoxication were shown. Although the accurate gas concentration inside his mask was unknown, as the mask was connected to a source supplying only nitrogen gas, we assumed that he inhaled an extremely high concentration of nitrogen gas. On the basis of these findings and the information regarding when the victim was found, the cause of death was diagnosed as nitrogen poisoning.

## Discussion

In cases of fatal occupational accidents, performing an autopsy offers several benefits. First, it helps to clarify the mechanism of death, including whether the death occurred suddenly or if there was potential for resuscitation. Next, internal contributing factors, such as medical history and comorbidities that may have influenced the death, as well as the involvement of substances including drugs, toxins, or alcohol were confirmed. By comprehensively integrating this autopsy data with findings from police investigations, more specific and detailed preventive measures can be developed. To our knowledge, the present case is the first case report presenting detailed forensic autopsy findings (external examination, internal findings, histopathology, and toxicology) of a worker who died from nitrogen poisoning while wearing a mask. We believe that the result obtained from our present autopsy case might contribute to the establishment of effective preventive measures. Giorgetti et al. reviewed fatal nitrogen inhalation cases in the English literature between 1984 and 2020 [9]. They searched PubMed with the terms “nitrogen” AND “death” OR “autopsy” OR “suicide” OR “accidental” OR “intoxication.” We further performed a similar search of PubMed until the end of 2024. However, no additional cases were found. Therefore, 24 decedents in 11 articles included in Giorgetti et al.’s review [9] were analyzed (Fig. 2). After excluding suicide cases (7 decedents in 4 articles), 17 unintentional deaths were recorded in 7 articles. Regarding the circumstances of nitrogen gas exposure, 12 decedents (6 articles) had inhaled nitrogen in a limited area, whereas 5 decedents had inhaled nitrogen via a mask similar to the present case (Fig. 2). We were able to obtain detailed circumstances of inhalation from the five decedents [7]. In these five cases, tubes of the mask had misconnected with the valve of the air line. Table 1 shows the cause of the misconnection. In three cases, discrimination between the air line and other gases was difficult because of mislabeling or no labels. In the present case, although the color of the label and name of the gas were not identical, the decedent had unintentionally connected the air line to the valve of a nitrogen gas bottle. The present case suggests that relying on visual information of one person is insufficient for preventing misconnection at the work place.

**Fig. 2:**
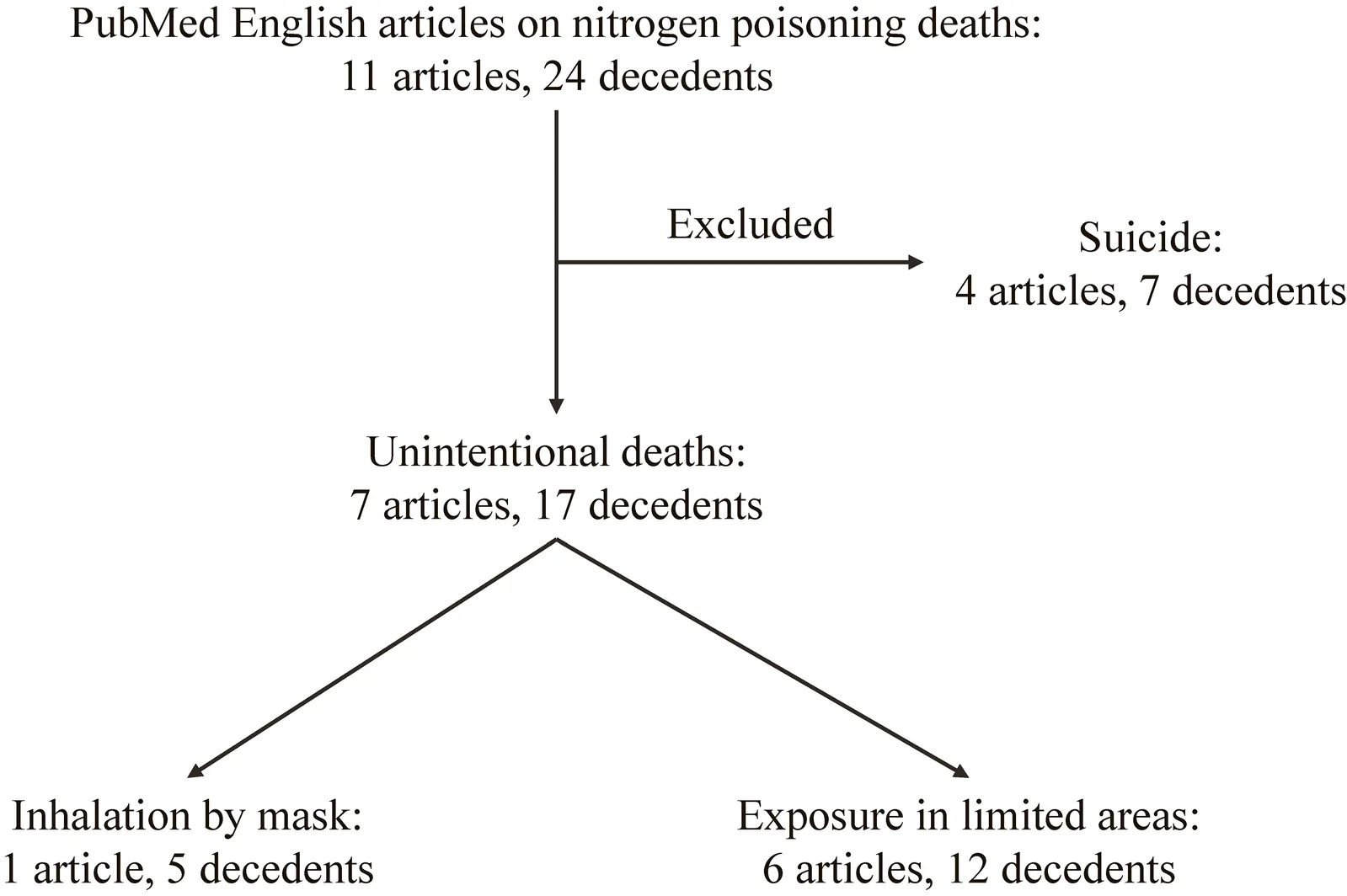
Review of fatal nitrogen inhalation cases in the English literature between 1984 and 2024.

**Table 1:** Cause of misconnection in five decedents who inhaled nitrogen by a mask (1984–1988) [7].

**No.**	**Age (y)**	**Occupation**	**Cause of misconnection**
1	34	Manufacturer	Same size and color and similar labeling*^1^
2	31	Painter	Mislabeled*^2^
3	56	Sandblaster	Unlabeled*^3^
4	33	Sandblaster	Unlabeled*^3^
5	22	Pourer	Not determined

*^1^Same size and color and similar labeling: the size and color of labeling of the air and nitrogen bottles were similar.

*^2^Mislabeled: the nitrogen and air line labels were incorrectly attached.

*^3^Unlabeled: the type of material supplied was not clearly labeled.

In Japan, fatal work-related accidents need to be reported to Ministry of Health, Labour and Welfare. According to their registration, work-related fatal nitrogen poisoning cases were extracted from 1993 to 2022 [10]. We found 32 decedents during these 30 years. After excluding two decedents who had aspirated complex gas (nitrogen, carbon monoxide, and carbon dioxide gases), four decedents had inhaled nitrogen gas via a mask, as in the present case (Fig. 3). However, in these cases, neither detailed circumstances at inhalation nor medical information was obtained.

**Fig. 3:**
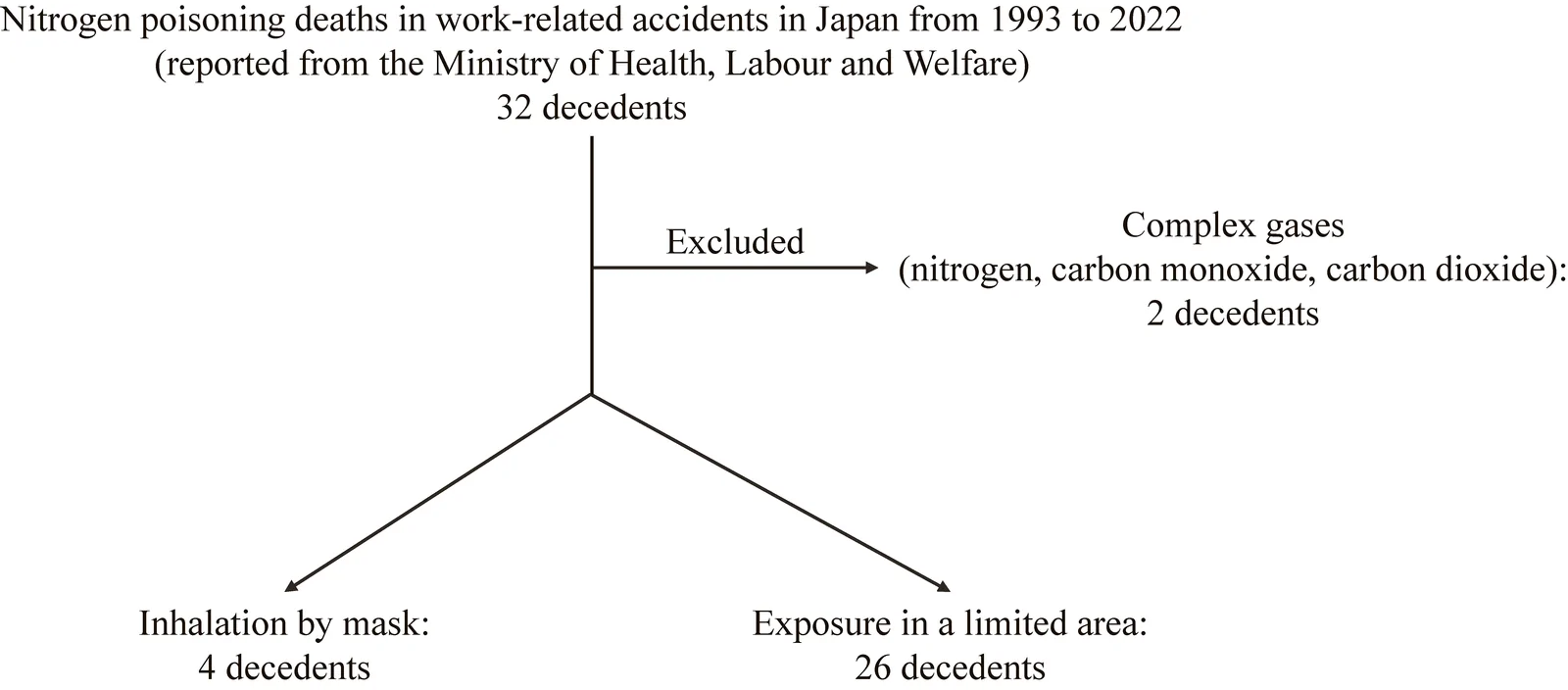
Review of work-related fatal nitrogen gas poisoning cases in Japan from 1993 to 2022.

We propose two potential preventive measures for fatal nitrogen gas inhalation during work based on our autopsy case and a review of the literature (Fig. 4). First, to actively prevent human errors, we now explicitly propose a mechanical preventive measure based on the circumstances of this case. Specifically, we implement fail-safe designs such as dedicated connectors for different gas lines (utilizing unique shapes or pin-index safety systems) and the strict separation of gas supply systems. Furthermore, as a conceptual safety strategy requiring further engineering validation, oxygen monitoring devices could be attached to workers’ masks and configured to automatically shut off the gas supply if the oxygen concentration drops below a safe threshold (such as the atmospheric threshold). Secondary, when wearing a mask and inhaling air or oxygen, workers should not connect an air line to the valve of a gas bottle alone, and double-checking the system should be performed. If a worker misconnects the air line to other gases, another worker may notice it and avoid fatal inhalation. If a worker inhales critical gases, such as nitrogen, another worker may notice it and remove the mask, and then resuscitation can be performed immediately. This is a simple but important preventive measure for fatal work-related accidents of incorrect gas inhalation. We believe these two preventive measures could help to reduce the risk of similar fatal occupational accidents.

**Fig. 4:**
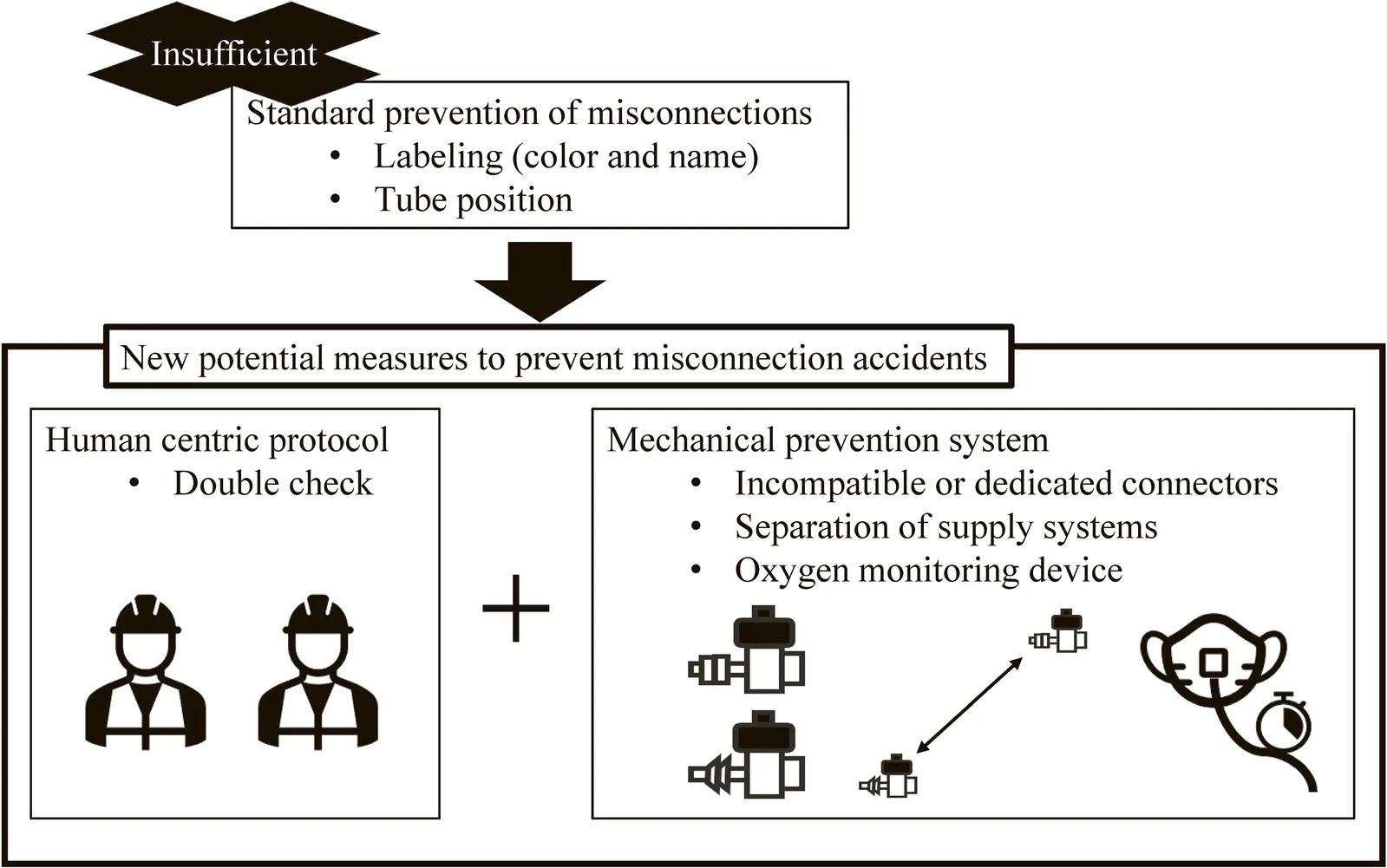
Our proposal of two potential preventive measures for fatal nitrogen gas inhalation during work.

## Conclusions

This case suggests that relying on a single operator’s visual verification is insufficient to prevent fatal mask misconnections in the workplace, although color-coded labeling is standard practice. To help prevent such tragic industrial accidents, we propose two potential preventive measures: several technical approaches for fail-safe designs, such as incompatible or dedicated connectors, separation of supply systems and equipping masks with oxygen monitoring devices that automatically shut off the gas supply when the oxygen concentration falls below a safe threshold, and implementing a strict two-person “double-check” system.

## Declarations

### Availability of data and material

Data are available on request from the corresponding author.
